# Under Pressure, but Together: Stressor Reactivity and Daily Social Connection in Middle and Later Adulthood

**DOI:** 10.1093/geroni/igaf122.637

**Published:** 2025-12-31

**Authors:** Susan Charles, Eric Cerino, Jennifer Piazza, David Almeida, Jonathan Rush

**Affiliations:** University of California, Irvine, Irvine, California, United States; Northern Arizona University, Flagstaff, Arizona, United States; California State University, Fullerton, Fullerton, California, United States; The Pennsylvania State University, University Park, Pennsylvania, United States; University of Victoria, Victoria, British Columbia, Canada

## Abstract

Social belonging has long been conceptualized and studied as a stable individual difference characteristic, shaped by temperament, personality, and childhood experiences. In this talk, we will discuss how feelings of social connection vary on a daily basis, and how these variations are related to stressor reactivity. Using eight days of daily diary data from the National Study of Daily Experiences (N = 2,022), we examine how daily social connection is related to negative affect experienced in response to a stressor in a national sample of midlife and later adulthood. Each day, participants reported whether they had experienced a daily stressor (e.g., an argument, a stressor at work, a stressor at home), and the extent to which they felt a sense of belonging across the past 24 hours. We used multilevel modelling to examine how levels of daily social belonging were related to changes in negative affect on days when people responded to stressors, after adjusting for average levels of positive affect, stressor exposure, and parental warmth. In a model examining the main effects, greater sense of belonging was related to lower levels of negative affect ( γ =-.022, p<.001), and stressor exposure was related to higher negative affect (γ =.127, p<.001). However, the addition of the within-person interaction indicated that negative affect was lower on stressor days when people felt a higher sense of belonging (γ = -0.065, p<.001). Results suggest that daily connection is important for well-being and how we respond to stressors in daily life.

